# Building National Health Security Through a Rapid Self-Assessment and Annual Operational Plan in Uganda, May to September 2021

**DOI:** 10.1089/hs.2022.0107

**Published:** 2023-04-03

**Authors:** Maureen Nabatanzi, Herbert Bakiika, Immaculate Nabukenya, Mohammed Lamorde, Justine Bukirwa, Martha I. Achan, Peter A. Babigumira, Lydia Nakiire, Timothy Lubanga, Enid Mbabazi, Roland B. Taremwa, Harriet Mayinja, Anne Nakinsige, Douglas K. Makanga, Allan Muruta, Solome Okware, Innocent Komakech, Issa Makumbi, Milton M. Wetaka, Joshua Kayiwa, Felix Ocom, Alex R. Ario, Sandra Nabatanzi, Joseph Ojwang, Amy Boore, Rahel Yemanaberhan, Christopher T. Lee, Ekwaro Obuku, Daniel Stowell

**Affiliations:** Maureen Nabatanzi, MPHN, FETP, is an Epidemiologist, Global Health Security Department, Infectious Diseases Institute, Kampala, Uganda.; Herbert Bakiika MPH, is a One Health Specialist, Global Health Security Department, Infectious Diseases Institute, Kampala, Uganda.; Immaculate Nabukenya, PhD, is an Epidemiologist, Global Health Security Department, Infectious Diseases Institute, Kampala, Uganda.; Mohammed Lamorde, MD, PhD, is Head of Department, Global Health Security Department, Infectious Diseases Institute, Kampala, Uganda.; Justine Bukirwa is a Technical Officer, Laboratory Systems, Global Health Security Department, Infectious Diseases Institute, Kampala, Uganda.; Martha I. Achan, LLB, LLM, is a Legal Advisor, Global Health Security Department, Infectious Diseases Institute, Kampala, Uganda.; Peter A. Babigumira, BPharm, is Technical Advisor, Emergency Preparedness and Response, Global Health Security Department, Infectious Diseases Institute, Kampala, Uganda.; Lydia Nakiire, MPH, FETP, is an Epidemiologist, Global Health Security Department, Infectious Diseases Institute, Kampala, Uganda.; Timothy Lubanga is Commissioner, Monitoring and Evaluation Department, Office of the Prime Minister, Kampala, Uganda.; Enid Mbabazi, MD, is a Medical Officer, Office of the Prime Minister, Kampala, Uganda.; Roland B. Taremwa is a Monitoring and Evaluation Specialist, Office of the Prime Minister, Kampala, Uganda.; Harriet Mayinja is a Research Officer, Integrated Epidemiology, Surveillance and Public Health Emergencies Department, Ministry of Health, Kampala, Uganda.; Anne Nakinsige, MD, is Principal Epidemiologist, Integrated Epidemiology, Surveillance and Public Health Emergencies Department, Ministry of Health, Kampala, Uganda.; Douglas K. Makanga, MD, is a Medical Officer, Integrated Epidemiology, Surveillance and Public Health Emergencies Department, Ministry of Health, Kampala, Uganda.; Allan Muruta, MD, MPH, is Commissioner, Integrated Epidemiology, Surveillance and Public Health Emergencies Department, Ministry of Health, Kampala, Uganda.; Solome Okware, MD, MPH, is an Epidemiologist, World Health Emergencies Cluster, World Health Organization, Uganda Country Office, Kampala, Uganda.; Innocent Komakech, MD, MPH, is an Emergency Readiness Officer, World Health Emergencies Cluster, World Health Organization, Uganda Country Office, Kampala, Uganda.; Issa Makumbi, MD, MSc, is Director, Public Health Emergency Operations Center, Ministry of Health, Kampala, Uganda.; Milton M. Wetaka is a Laboratory and Logistics Specialist, Public Health Emergency Operations Center, Ministry of Health, Kampala, Uganda.; Joshua Kayiwa, MSc, is a Data Analyst, Public Health Emergency Operations Center, Ministry of Health, Kampala, Uganda.; Felix Ocom, MD, is Deputy Manager, Public Health Emergency Operations Center, Ministry of Health, Kampala, Uganda.; Alex R. Ario, MD, PhD, is Managing Director, Uganda National Institute for Public Health, Ministry of Health, Kampala, Uganda.; Sandra Nabatanzi, MSC, is an Emergency Management and Response Specialist and Outbreak Coordinator, Division of Global Health Protection, US Centers for Disease Control and Prevention Uganda Country Office, Kampala, Uganda.; Joseph Ojwang, MD, MPH, is a Public Health Specialist, Division of Global Health Protection, US Centers for Disease Control and Prevention Uganda Country Office, Kampala, Uganda.; Amy Boore, MD, PhD, is Director, Division of Global Health Protection, US Centers for Disease Control and Prevention Uganda Country Office, Kampala, Uganda.; Rahel Yemanaberhan, MSc, is Regional Technical Advisor (East Africa), Resolve to Save Lives Ethiopia Country Office, Addis Ababa, Ethiopia.; Christopher T. Lee, MD, MPH, is Director of Global Preparedness and Response, Resolve to Save Lives, New York, NY.; Ekwaro Obuku, MD, PhD, is Senior Technical Advisor, Data and Policy, Global Health Security Department, Infectious Diseases Institute, Kampala, Uganda.; Daniel Stowell, MPH, is a Global Health Security Specialist, US Centers for Disease Control and Prevention, Atlanta, GA.

**Keywords:** Self-assessment, Operational planning, Public health preparedness/response, Joint External Evaluation, National Action Planning for Health Security, International Health Regulations

## Abstract

Uganda established a National Action Plan for Health Security in 2019, following a Joint External Evaluation (JEE) of International Health Regulations (2005) capacities in 2017. The action plan enhanced national health security awareness, but implementation efforts were affected by limited funding, excess of activities, and challenges related to monitoring and evaluation. To improve implementation, Uganda conducted a multisectoral health security self-assessment in 2021 using the second edition of the JEE tool and developed a 1-year operational plan. From 2017 to 2021, Uganda's composite ReadyScore improved by 20%, with improvement in 13 of the 19 technical areas. Indicator scores showing limited capacity declined from 30% to 20%, and indicators with no capacity declined from 10% to 2%. More indicators had developed (47% vs 40%), demonstrated (29% vs 20%), and sustained (2% vs 0%) capacities in 2021 compared with 2017. Using the self-assessment JEE scores, 72 specific activities from the International Health Regulations (2005) benchmarks tool were selected for inclusion in a 1-year operational plan (2021-2022). In contrast to the 264 broad activities in the 5-year national action plan, the operational plan prioritized a small number of activities to enable sectors to focus limited resources on implementation. While certain capacities improved before and during implementation of the action plan, countries may benefit from using short-term operational planning to develop realistic and actionable health security plans to improve health security capacities.

## Introduction

The health and economic impact of the COVID-19 pandemic has illustrated why every country must improve its ability to detect and rapidly respond to deadly infectious disease outbreaks. Uganda is prone to infectious disease threats and frequently experiences outbreaks of measles,^1^ typhoid,^2^ anthrax,^3^ yellow fever,^4^ malaria,^5^ cholera,^6^ viral hemorrhagic fevers,^7^ and avian influenza.^8^ Over the last 6 years, Uganda has accelerated investments in strengthening outbreak detection and response capacity toward standards set under the 2005 International Health Regulations (IHR).^9^

During this time, Uganda served as a founding member of the Global Health Security Agenda (GHSA) and hosted countries to extend the GHSA initiative for 5 more years through the Kampala Declaration.^10^ Uganda initially piloted the GHSA assessment in 2015^11^ and conducted its Joint External Evaluation (JEE) in 2017.^12^ In addition, Uganda complies with other requirements of the IHR *Monitoring and Evaluation Framework*,^13^ including State Party Self-Assessment Annual Reporting (SPAR),^14^ simulation exercises, and after-action reviews.^15^

The second edition of the JEE tool provides a thorough review and scoring of a country's capacities in 19 technical areas of public health.^16^ The JEE also helped establish new norms for country-led planning, transparency, and set standard definitions in global health security.^17^ In addition to individual technical area assessment scores, countries can use a composite average across all areas to provide a higher-level overview of national readiness. For example, ReadyScore, developed by Resolve to Save Lives, is an average score of the 19 technical areas.^18^ Uganda's 2017 JEE revealed a critical need to expand multisectoral coordination and strengthen capacities in surveillance, national laboratory systems, emergency response operations, points of entry, and national legislation.^12^ Following the JEE, Uganda initiated the development of a 5-year National Action Plan for Health Security (NAPHS) to address JEE recommendations and define strategies and activities for improvement.^15^ The NAPHS was launched in 2019 with 264 activities across the 19 technical areas aligned with top government and sectoral priorities. The NAPHS enabled joint multisectoral planning for health security in a structured way, which contributed to successful preparedness and response efforts to concurrent public health emergencies.^8,19^ However, the country had challenges implementing the corrective actions following the planning. This was due to the bulk of activities in the NAPHS lacking adequate funding, accountability, and monitoring required to drive whole-of-government health security activities forward. By 2020, only 40% of NAPHS activities were funded and 9% of NAPHS activities had completed implementation.

In May and June 2021, Uganda conducted a health security self-assessment using the second edition of the JEE tool^16^ to identify interim progress made and prioritize capacity building actions in a 1-year operational plan for October 2021 through September 2022. In this article, our aim is to share Uganda's experience with the self-assessment and 1-year operational plan, as other countries may find it a valuable process to supplement the JEE.

## Methods

The JEE self-assessment and operational planning began in February 2021 with internal consultations with the Office of the Prime Minister, which leads NAPHS coordination and monitoring and evaluation of all ministries, departments, and agencies. Following approval, the Office of the Prime Minister sent official invitations to the ministries, departments, agencies, and organizations to nominate participants. Implementation of IHR in Uganda is multisectoral and multidisciplinary, involving government, private, and development partners. Forty-two organizations across 11 sectors were purposively selected to nominate IHR technical area subject matter experts to participate, including national IHR focal persons. Table 1 shows the number of participants by sector and organization. In total, 182 people participated in the assessments; 110 (60%) were from human health and 25 (14%) were from the animal health and agricultural sectors.

**Table 1.** Sectors, Ministries, Departments, Agencies, Organizations, and Participants During Uganda's Self-Assessment of Health Security in 2021

Sector	Name of Ministry, Department, Agency, Organization	Number of Entities	Number of Participants
Human health	Amref Health Africa; Baylor College of Medicine Children's Foundation-Uganda; US Centers for Disease Control and Prevention; Central Public Health Laboratories; Infectious Diseases Institute; Makerere University School of Public Health; Makerere University Walter Reed Project; Médecins Sans Frontières; Ministry of Health; Public Health Emergency Operation Center; Resolve to Save Lives; Uganda Public Health Fellowship Program; Uganda Red Cross Society; Infectious Disease Detection and Surveillance project; Uganda Virus Research Institute; World Health Organization	16	110
Animal health and agriculture	Africa One Health University Network; United Nations Food and Agriculture Organization; Food Safety Associates Limited; Ministry of Agriculture, Animal Industry and Fisheries; Makerere University College of Veterinary and Biosecurity; United Nations World Food Programme	6	25
Social development	Ministry of Gender, Labour, and Social Development; Touched Souls Uganda; Uganda Consumers Protection Association; United Nations Children's Fund; United States Agency for International Development Social and Behavior Change Activity	5	11
Governance	District Health Office; Kampala Capital City Authority; Office of the Prime Minister; Presidential Scientific Initiative on Epidemics, Statehouse Uganda	4	9
Wildlife and tourism	Conservation Through Public Health; Ministry of Tourism, Wildlife and Antiquities (Uganda Wildlife Authority)	2	7
Environmental health	Ministry of Water and Environment; National One Health Platform	2	5
Security and defense	Ministry of Defence and Veterans Affairs (Uganda People's Defence Force); Ministry of Security (Uganda Police Force)	2	5
Internal affairs	Ministry of Internal Affairs, Directorate of Government Analytical Laboratory	1	5
Science and technology	Ministry of Science, Technology and Innovation; Uganda National Council for Science and Technology	2	3
Justice	Ministry of Justice and Constitutional Affairs	1	1
Energy	Uganda Atomic Energy Council	1	1
Total		42	182

### Facilitator Training

On May 17, 2021, the Ministry of Health held an introductory training at the Public Health Emergency Operation Center (PHEOC) to orient facilitators to the JEE tool and assessment and planning process. A total of 92 participants attended either physically or online via Zoom in light of COVID-19 social distancing recommendations. Fifteen of the trained participants were selected as lead facilitators for the technical area discussions. Lead facilitators were selected based on their expertise in multiple technical areas, experience gained during the 2017 JEE, and participation in SPARs.

### Online Meetings by Technical Area

Over the period of a week (May 25 to 28, 2021), 19 meetings for each of the technical areas were held to determine the JEE capacity level scores. Meetings were held at the PHEOC and online. Each meeting was chaired by a representative from the Office of the Prime Minister, led by a facilitator and attended by multisectoral participants. The self-assessment and scoring exercise by technical area were guided by a Microsoft PowerPoint presentation template. Participants reviewed the 2017 JEE report,^12^ NAPHS,^15^ after-action review reports, preparedness and response plans, sector strategic plans, sector performance reports, benchmarks,^20^ and other supportive guidelines and policies to identify strengths and gaps for each indicator. Participants then held in-depth discussions, shared expert opinions, documented the progress made since 2017, updated the score using the JEE tool, and provided the rationale for the score. For both 2017 and 2021, we computed the ReadyScore for health security, an overall average score for the 19 technical areas, using this formula:^18^

$$\begin{array}{c} ReadyScore=\\ \left(\frac{Overallaveragescoreofthe19technicalareaaverages}{5}\right)X100 \end{array}$$

The ReadyScore represents a composite average across all technical areas, with a maximum score of 100%. Higher ReadyScores represent a higher degree of preparedness for epidemics and other health security threats.^18^ Participants also identified 1 to 2 critical benchmark activities per technical area to form an operational plan that would be completed in the next year. Activities were assigned points of contact, timelines, and a proposed source of funds for implementation. Meetings lasted 1 to 3 hours, depending on the size and complexity of the technical area.

### Consensus and Prioritization Meeting

Following the self-assessment meetings, an editorial team consisting of staff from the Ministry of Health and the Office of the Prime Minister collated workshop notes and compiled a report. In June 2021, the report was disseminated to participants for review. A 2-day consensus meeting was held on September 22–23, 2021 where top-level government and partner officials approved of the scores and prioritized activities in the operational plan. The final assessment report with the operational plan was disseminated to ministries, departments, and agencies to guide health security implementation.

### Post-Assessment and Operational Planning Survey

On October 3, 2021, we conducted a process evaluation to determine Uganda's operational planning workshop participants' perspectives on whether the process had contributed to health security implementation in Uganda. A survey sent to participants via email assessed the following 3 indicators: Uganda's ability to (1) determine health security capacities, (2) select activities to improve capacities in technical areas and in turn JEE scores, and (3) address health security gaps.

### Ethical Statement

This activity was conducted to assess Uganda's health security capacities and was therefore determined to be nonresearch. The Office of the Prime Minister of the government of Uganda gave the directive and approval to conduct this activity. This activity was not human subject research, did not involve individual person identifiers, and its primary intent was to inform public health practice.

## Results

### Changes in Uganda's Joint External Evaluation Scores

The health security self-assessment revealed improvements in Uganda's ReadyScore from 50% in 2017 to 60% in 2021—a 20% increase. In 2021, 13 technical areas registered average improvements in health security capacity across indicators, whereas 6 technical areas had no average change in capacity across indicators (Table 2). Uganda increased its capacity in 17 indicators (35%), had no change in capacity in 31 indicators (63%), and reduced capacity in 1 indicator (2%) (Table 3). Specifically, of the 49 indicators, Uganda had more indicators with sustainable (n=1, 2%), demonstrated (n=14, 29%), and developed (n=23, 47%) capacity compared with limited (n=10, 20%) and no (n=1, 2%) capacity (Table 3). Sustainable capacity for an indicator in immunization was due to the availability of vaccine delivery in all districts. An indicator in emergency preparedness had no capacity because a draft national multihazard preparedness and response plan was not finalized.

**Table 2.** Change in Uganda's JEE Capacity Scores by Technical Area, 2017 to 2021

Change	Technical Area	Number of Technical Areas
Increased	National Legislation, Policy, and Financing	13
	International Health Regulations (2005) Coordination, Communication, and Advocacy	
	Antimicrobial Resistance	
	Zoonotic Diseases	
	Food Safety	
	Immunization	
	National Laboratory System	
	Human Resources	
	Emergency Preparedness	
	Medical Countermeasures and Personnel Deployment	
	Risk Communications	
	Points of Entry	
	Radiation Emergencies	
No change	Biosafety and Biosecurity	6
	Surveillance	
	Reporting	
	Emergency Operations and Response	
	Linking Public Health and Security Authorities	
	Chemical Events	

Abbreviation: JEE, Joint External Evaluation.

**Table 3.** Changes in Uganda's JEE Scores by Technical Area and Indicator, 2017 to 2021

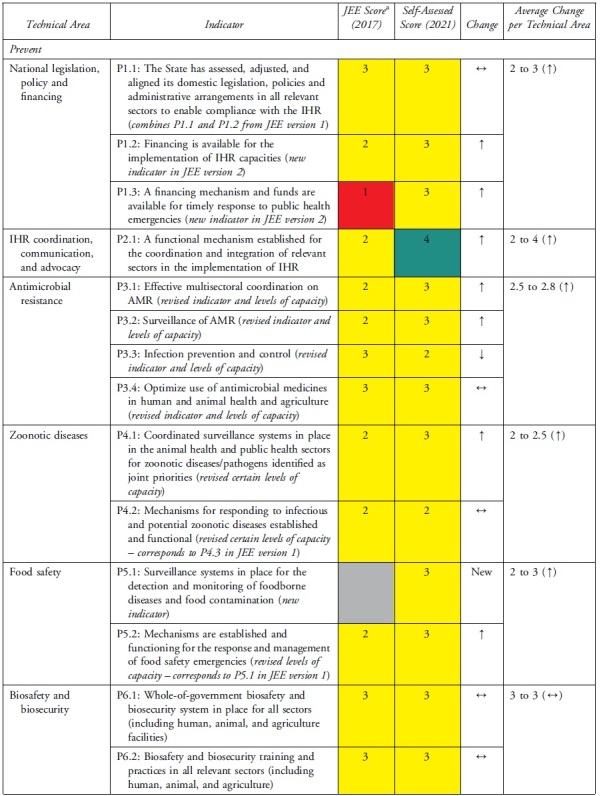 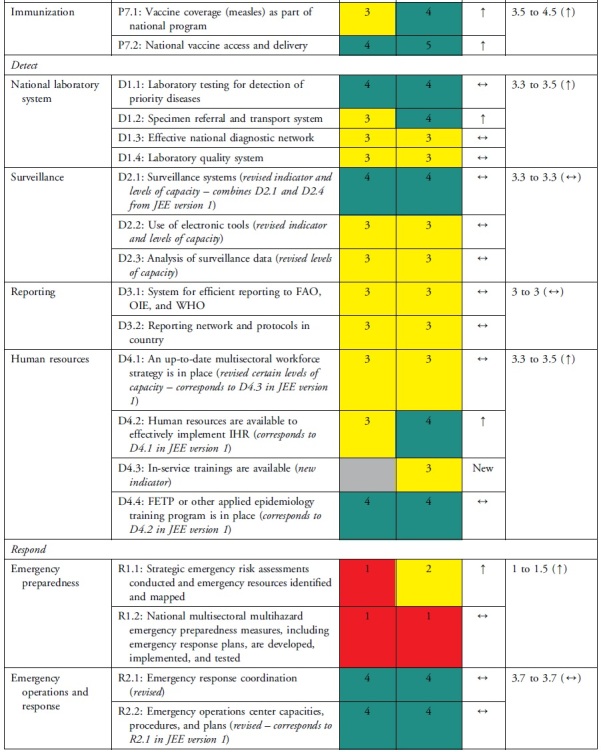 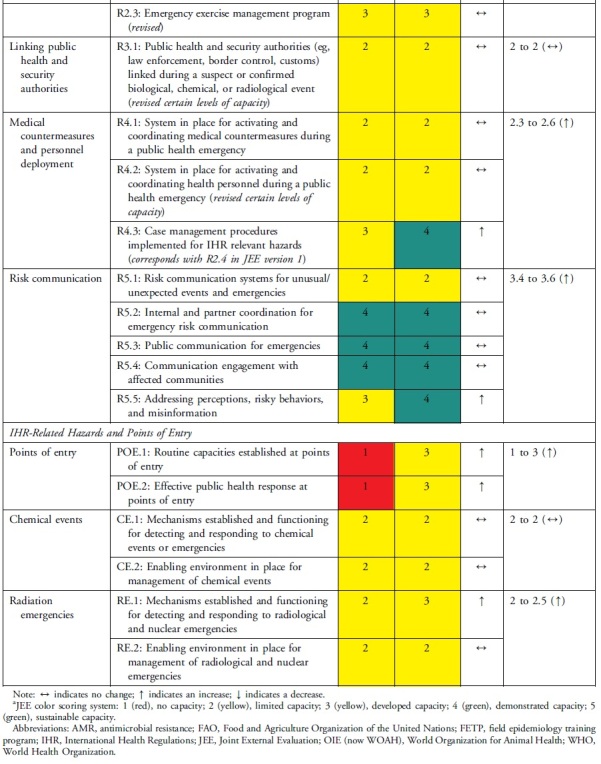

### Technical Areas Where Capacity Scores Increased

National legislation, policy, and financing and points of entry—recommended as priority areas by the 2017 JEE—had 3 indicators (P.1.3, POE.1, POE.2) that improved by 2 capacity level scores. P.1.3 improved due to amendments to Uganda's Public Health Act^21^ enacted in 1935 to domesticate IHR. Capacity at points of entry improved following Uganda's designation of 3 points of entry, establishment of a National Border Health Unit at the Ministry of Health, and investment in infrastructure and resources during the COVID-19 pandemic. The health sector can request to access the national contingency fund during a public health emergency, if required. Risk communication improved through rumor monitoring and public engagement to dispel misinformation during the COVID-19 response. IHR coordination, reporting, and linking public health and security improved by designation of official focal points.

### Technical Areas Where Capacity Scores Stagnated

Not all increases in indicator capacity scores were noteworthy improvements in the respective technical areas. Medical countermeasures increased in indicator R.4.3 but did not change in 2 indicators (R.4.1 and R.4.2). Emergency preparedness increased in indicator R.1.1 but stagnated in R.1.2 because of incomplete multihazard plans. The areas of antimicrobial resistance, chemical events, and radiological emergencies maintained low scores because the plans, guidelines, legislation, or other draft agreements were not finalized. In antimicrobial resistance, 2 indicators (P.3.1 and P.3.2) increased while indicator P.3.4 did not change. Due to changes in the second edition of the JEE tool, Uganda showed a decrease in capacity for the antimicrobial resistance indicator P.3.3, which changed from healthcare-associated infection prevention and control programs to include farms. Implementation of infection prevention and control at animal farms is still in its infancy. Radiation emergencies increased score in indicator R.E.1 but did not change in indicator R.E.2. None of the scores for the indicators in chemical emergencies increased.

### Results of Operational Planning

During operational planning, we developed a 1-year plan with 72 activities (mean per technical area = 3) compared with 264 activities (mean per technical area = 13) in the NAPHS. Each technical area had specific prioritized activities, authorities, and focal person(s) responsible for implementation, timeline, and a proposed source of funds.

**Figure 1.**
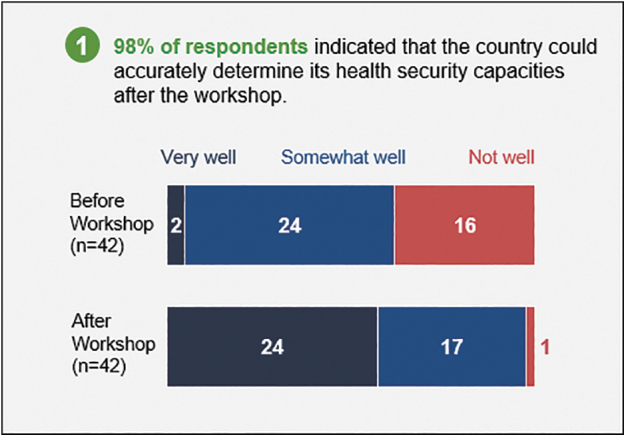
Survey results on Uganda s ability to determine health security capacities before and after the self-assessment and operational planning workshop.

**Figure 2.**
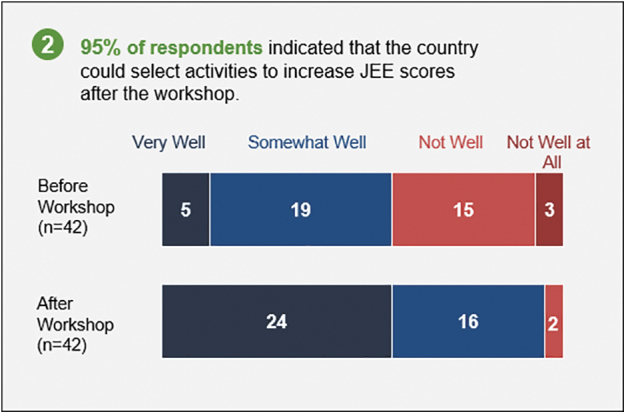
Survey results on Uganda s ability to select activities to increase health security capacity and JEE scores before and after the self-assessment and operational planning workshop

The online post-assessment and operational planning survey received 42 (23% of 182 participants) responses representing the government of Uganda and partners. Of these respondents, almost all (98%) indicated that the country could accurately determine its health security capacities after the workshop vs 62% before the workshop (Figure 1). After the workshop, 95% of the respondents indicated that the country could select activities to improve capacities in technical areas, and in turn JEE scores, following the workshop vs 57% before the workshop (Figure 2). All (100%) respondents indicated that activities selected during the workshop will address health security gaps over the next 12 months.

## Discussion

After experimenting with different approaches to health security assessment and planning, Uganda has uncovered key lessons on how to strengthen the evaluation process in improving health security capacities. Since the JEE in 2017 and NAPHS development in 2019, Uganda made notable improvements in health security capacity.

During the NAPHS development, Uganda selected activities to prioritize for implementation using a whole-of-government and One Health approach.^15^ The government received funds from partners including the US Centers for Disease Control and Prevention, World Health Organization, and Resolve to Save Lives to strengthen core capacities in surveillance, points of entry, laboratory systems, workforce development, emergency management, and legislation.^22-24^ Uganda set up a NAPHS acceleration project team within the Ministry of Health to work with relevant sectors to jumpstart implementation and coordinate and monitor NAPHS using a digital dashboard on Airtable (Airtable, San Francisco, CA)—a platform for building connected applications.^25^ This combination of prioritization, identification of funds, and human resources to implement activities resulted in improvements in difficult areas such as points of entry.

The COVID-19 pandemic accentuated the need for robust health security. Uganda leveraged COVID-19 national and international funding and made investments in infrastructure, capacity building, and human resources that resulted in improved capacities. Specifically, points of entry benefited from improved screening centers with dedicated personnel and information technology.^26^ In the legislation technical area, acceleration in the amendment of the Public Health Act and Rules^21^ was necessary to enable COVID-19 laws restricting travel and public gatherings. Similar to Uganda, in neighboring East Africa, supportive legislation enabled enactment of laws on cross-border and in-country travel restrictions, lockdowns, and closures for COVID-19 prevention.^27-33^ The self-assessment reflects contributions from COVID-19 investments that bridged longstanding gaps in national health security.

However, resource allocation has not always included activities needed to address bottlenecks to improving capacity. A 2021 review of Uganda's NAPHS revealed that while 42% of activities were partially funded and 40% were fully funded, 17% were not funded.^34^ To enhance health security, the government should provide guidance for implementers and partners to align operational plans and NAPHS with preidentified country priorities.

The self-assessment and operational planning were led by the Office of the Prime Minister, which helped mobilize high-level government participants, implementing partners, and nongovernmental organizations. Multisectoral engagement enriched the assessment and prioritization process. In Uganda, the Office of the Prime Minister and the Ministry of Health have also provided stable political and multisectoral support for health security during the GHSA launch in 2014,^11^ JEE in 2017,^12^ and NAPHS in 2019.^15^ Nigeria used a similar approach that involved joint identification of gaps and solutions to ensure collective contribution.^35^ This multisectoral demonstration of interest in health security activities should be leveraged to improve sector advocacy for inclusion of activities in sector budgets and plans to foster implementation.

Uganda followed global guidance for countries to describe 5-year priorities, costs, and to have an implementation plan after JEE.^36^ However, this was a lengthy process and these efforts were difficult to combine effectively. Therefore, Uganda's resulting 5-year NAPHS contained many activities with multiple subactivities and unfunded mandates. By 2021, the third year of NAPHS implementation, a mere 20% of activities planned were completed.^34^ Uganda and Nigeria have indicated that bulky national action plans for health security are difficult to track even with electronic monitoring tools.^35,36^ In our experience, multisectoral self-assessment operational planning that involves government leadership, selection of a small number of realistic activities, and monitoring activities routinely can strengthen capacity. Our findings are corroborated by Nigeria's recommendation to conduct country-led self-appraisals to develop feasible IHR action plans.^35^

Self-assessments can be subject to self-reporting bias. To counter this, Uganda applied the second edition of the JEE tool, involved multiple IHR sectors and a peer consensus approach to review capacity and identify sector-specific areas for improvement. Uganda applied lessons learned from Nigeria and Liberia; both conducted interim self-assessments in 2019 using the JEE approach. Since then, both countries have cemented stakeholders' commitment to IHR through implementation of a Regional Disease Surveillance System Enhancement project.^35,37^ In terms of next steps, Uganda's sectors will collaborate to implement the operational plan, with periodic reviews to ensure all planned activities receive adequate attention and resources.

## Conclusion

Uganda piloted a flexible methodology for operational planning including tools to develop short-term realistic and actionable plans. This operational planning takes the strategic goals from the NAPHS and uses the World Health Organization benchmarks for IHR to plan a small number of short-term activities. This self-assessment demonstrated Uganda's successful health security capacity building efforts since 2017. Although progress stalled in some technical areas, this assessment identified activities that should be prioritized for implementation to strengthen capacity. In addition to the IHR monitoring and evaluation framework, this process can assist low- and middle-income countries to reprioritize opportunities for health security acceleration. We recommend interim self-assessments as a tool for multiple sectors to reprioritize activities and lobby for projects and funds that improve health security capacity.
